# Collectively playable wearable music: Practice-situated approaches to participatory relational inquiry

**DOI:** 10.1017/wtc.2021.19

**Published:** 2022-02-24

**Authors:** Seth D. Thorn, Halley L. Willcox

**Affiliations:** 1 School of Arts, Media and Engineering, Arizona State University, Tempe, Arizona, USA; 2 School of Music, Dance and Theatre, Arizona State University, Tempe, Arizona, USA

**Keywords:** co-design, interactive dance, movement and computing, movement sonification, music, practice-situated research, real-time interactive media, telematic music, wearables, workshopping participation

## Abstract

We present two practice-situated participatory investigations using networked wearable sensors to develop movement-responsive collectively playable musical instruments: a series of four collocated workshops for expert dancers and a distance learning course in which students use wearable technology to enhance embodied learning and feelings of connectedness telematically. We reflect on our exploration of techniques for structuring ensemble improvisations augmented with bespoke digital musical instruments using aggregate statistical measures, such as variance of participants’ physical orientation as an index of group intention. Participatory design exchanges top-down design methodologies with bottom-up approaches consulting actors’ interests. We follow this approach by evolving our instruments through abductive experiments and trial-and-error tinkering, without strong theories, methods, or models, using elementary signal processing techniques that are meaningfully understood and modified by participants. Our experiences suggest useful scaffolding techniques for educational transdisciplinary research-creation communities seeking to explore relational ensemble dynamics in telematic and/or physically collocated settings using accessible wearable technologies. Through creative inquiry and participation, technical objects can become bearers of sense and meaning rather than instating mystifying or alienating relations for the participants.

## Introduction

Less cost-prohibitive wearable technologies have made once rarefied digital possibilities for movement and computing (MOCO), especially arts-based practice and inquiry, widely accessible. In this article, we describe our work with musical and movement-responsive networked wearable sensors whose collective sonic response we design together with epistemically diverse participants to influence group improvisation dynamics. An early precedent is the artistic collaboration of John Cage and Merce Cunningham in *Variations V*, a piece in which photocells and the capacitive response of radio antennas are used to turn a stage into a collectively playable, continuously responsive instrument (Nyman, 1999). Networked wearable computing invigorates the possibilities for creating *less spatially bounded* and *less individualistic*, collaborative, room-scale digital musical instruments (DMIs). We explore this potential by utilizing statistical aggregates of orientation and movement across multiple wearable sensors for parameter mapping sonification and auditory augmented feedback. This work is broadly motivated by improvisatory and relational movement practices and technology-augmented dance (e.g., Aylward et al., 2006; Naccarato and MacCallum, 2016; Himberg et al., 2018).

**Figure 1.**
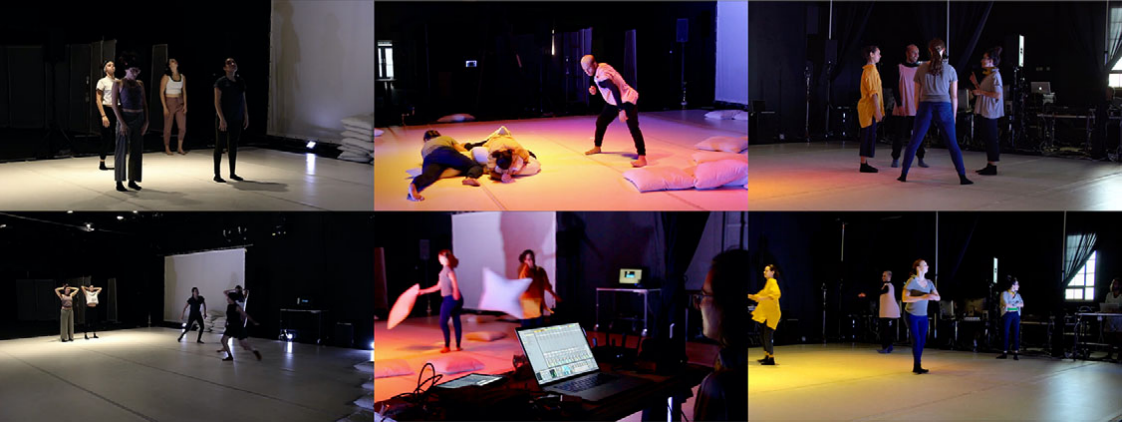
*Top left:* dancers instrumented with headband sensors. *Bottom left*: a non-instrumented dancer introducing a novel movement motif. *Center:* realization of a concise score with collectively playable wearable digital musical instruments (DMIs). *Top right:* second author (dancer and choreographer) in discussion with participants. *Bottom right*: she consults with the first author (managing sound design). Related videos can be found in an online Vimeo showcase (https://vimeo.com/showcase/8623644) and are individually referenced and linked throughout this article. (This collection is also permanently available at https://doi.org/10.5281/zenodo.5762453.)

Through a hybrid of bottom-up participatory design, sonic augmentation of movement using wearable computing, and experiential research in group dynamics, we creatively explore how configurations of movement-responsive auditory feedback affect a sense of togetherness in ensembles. Participatory design is a well-known design approach that self-consciously engages with the milieu of industrial democracy. By involving future users of a technology with shaping design processes in beneficial ways, while also addressing ethical and political implications, participatory design magnifies questions of democracy and power in workplaces (Ehn, 1993). Similarly, our investigation of epistemically diverse collective creativity in educational environments moves toward symmetrizing power relations, potentiating more equitable and novel outcomes. A characteristic feature is the lack of well-articulated problems, since these only become concrete during the actual experiments. We arrive, in other words, in a multifaceted space with wearable instruments, a diverse set of actors, and an inchoate set of ideas and intuitions about what we want to achieve, but without a blueprint for how to proceed (Figure 1). The way forward, here, is a process of “co-design,” a term that invokes a design situation in which problems and solutions co-evolve through collaborative effort (Steen, 2013). In such situations, where not even the problems are well-defined, abductive reasoning is employed, advancing by iteratively framing problems and potential solutions (Steen, 2013). A technocritical dimension of our work, likewise, is to foster a participatory technoculture in which actors *adapt* wearable technologies through process-based “embodied sensemaking”—first-person experiences activating and enlivening the abductive process (Hummels and Van Dijk, 2015). By contrast, a less critical and less embodied approach would tend to veil rather than expose the normative biases designed into wearables, to wit, measurement of productive but not “joyful” activity (Spiel et al., 2018). Technical objects do not just form a system of utility, but also generate sense and meaning as objects of culture. Thus, insofar as we combine academic research and artistic practice, our collective inquiry constitutes a “research-creation” event, an experimental approach that catalyzes the generation of new experiences and evolution of collective expression through diverse practices. As dancer and theorist Erin Manning notes, the knowledge this generates may be “extra-linguistic” and/or escape normative modes of inquiry (Manning, 2016). Nevertheless, we will do our best to articulate this knowledge to the reader by recapitulating our process concretely, in order to remain critically attentive to those “material complexities of participation” that resist the pretense of a de facto participatory situation wherever an assemblage of diverse actors, epistemes, and techniques is brought together (Tanaka and Parkinson, 2018). Bottom-up attention to process, in other words, acknowledges that participation is a contingent outcome whose success hinges on felicitous adaptation, exchange, and learning. There is no procedure to guarantee that success.

Our use of the term “assemblage” derives from Deleuze and Guattari, frequently cited philosophers in qualitative research methodologies (e.g., Barrett and Bolt, 2007; Koro-Ljungberg, 2015). Accordingly, an assemblage is a multiplicity of elements without a template, center, or hierarchy. Assemblages are never fixed; they continuously vary and evolve by making new connections (Deleuze and Guattari, 1987). In this article, we attempt to flesh out the concrete, fine, artistic and technical decisions that both shape, and are shaped by, these connections in our explorations of collective sonic interaction with wearables. We discuss our participatory approaches and distill principles for communities interested in this transdisciplinary research-creation. The two settings we recount are a series of four collocated workshops for dancers and a distance learning course for undergraduate students who explore embodied telematic togetherness using musical wearables. Given our collaboration with expert dancers—and the adept movement skills and embodied practices that they bring to the events, which includes the second author—we can further specify this work as a form “somatic connoisseurship” (Schiphorst, 2011). The rich movement histories and expertise of the dancers suffuse the workshops with tacit embodied knowledge. A rather different goal manifests in the wearable music class, which is to increase the corporeal engagement of nonspecialist students involved in distance learning by having them explore movement-driven sound, diminishing reliance on screen-based interactions. In both scenarios, we follow a pragmatic process-based approach attentive to phenomena emerging during these experiments, which reflects our commitment to “practice-situated” inquiry (Shotter, 2014). We address this approach in the following section before proceeding to descriptions of our experiences in the workshops and distance learning course. This paper extends a previous conference paper (Thorn et al., 2020) on the wearable music workshops by introducing many new reflections and details. It also extends a conference paper published by the first author (Thorn, 2021b) on the telematic wearable music class by adding new details, distilling specific design principles, and drawing connections between the development of that course and the knowledge and techniques it inherited from the dance workshops.

## Relational Experiential Inquiry in Movement Arts

### Practice-Situated Inquiry

Practice-situated “systemic” inquiry begins in the turbulent flow of ongoing activity, without ideally prepared conditions, grounding definitions, strong theories, or firm predictions (Shotter, 2014). One does not cease to plan and strategize, but structures must be fluid enough to respond to unforeseen (*im-pro-vised*) possibilities, which may be highly significant and show up by chance or intuition. Within the complex entanglement of bodies and worlds, practice-situated approaches bracket hard distinctions between subjects and objects, leaving these as outcomes rather than presuppositions of experiments (James, 1912). Attention is focused on the horizon of relation rather than vertical theory (*theoria*: “to behold”). In collective dance improvisation (CDI), leading and following are dynamical such that, in successful conditions, dancers describe synchronous continuity of moving and being moved (Sheets-Johnstone, 2011). “As we move together, the boundaries between moving and being moved blur” (Himberg et al., 2018). In taking this phenomenological description at its word, which is out of keeping with linear scientific-grammatical categories of cause/effect and subject/object, practice-situated approaches become attuned to the emergence of novel experiences. This style of inquiry remains receptive to contingencies and unexpected detours, those forces “beyond” the experiments—tight boundaries around which we do not willfully design or practice, as when an observer of an ensemble event suddenly leaps in to resolve a perceived tension in CDI. This ethos is prominent in experiential and situated third wave HCI, which loosens up formerly strict designer-user dichotomies (Williams and Irani, 2010).

### Intentionality in Relational Time-based Arts

Dance and music are time-based performative artistic traditions with a long historical affinity. Both are embodied, participatory, and relational practices. In the case of music:

[Music] is not a unidirectional process, with participants entraining to a particular individual who is the time-keeper; it is likely to involve a process of continuous reciprocal adaptation of the periods and phases of the sounds and actions produced… with each participant continually switching between leading and following each other. (Cross, 2014).

Contact improvisation, likewise, which is a style of dance that grew out of the zeitgeist of the 1960s, is a dramatic and rich relational practice:

Contact improvisation is most frequently performed as a duet, in silence, with dancers supporting each other’s weight while in motion. Unlike wrestlers, who exert their strength to control a partner, contact improvisers use momentum to move in concert with a partner’s weight, rolling, suspending, lurching together. They often yield rather than resist, using their arms to assist and support but seldom to manipulate. Interest lies in the ongoing flow of energy… (Novack, 1990)

In contact improvisation, the rolling point between bodies cannot be reduced to an aggregate of partners’ intentions. The cognitivist overtones of the word “intention,” suggesting reflexive mental planning prior to initiating an action, produce an unremarkable description of the ongoing flow in CDI, music, and other collective improvisatory practices (Angelino, 2018; Angelino, 2020). Noncognitivist formulae for embodied intentionality, such as “directing-itself-towards” (Heidegger, 1985) or “motor intentionality” (Merleau-Ponty, 1962),^1^ are more experientially faithful descriptions of CDI: these formulae imply that one need go no further than movement *tout court* to ascertain embodied intention.^2^ In CDI, the upshot is that the ensemble experience of togetherness is “kinaesthetic” (Himberg et al., 2018). Experiments using hypercomplex signal correlation and probabilistic measures of entropy have been explored to test this “connectedness” empirically (Krzyzaniak et al., 2015). This approach links to precedent work in social psychology connecting movement coordination and sociality, which observes that “interactions often have an affective dimension in the sense that we can feel varying degrees of connection with the other” (De Jaegher and Di Paolo, 2007). In our workshopping, “connectedness” or “togetherness” are not so much phenomena we seek to measure as ones we desire to enact, a corollary of our process-based approach. By way of example, consider the remark from one of our workshop participants that the sound generation associated with group orientation variance allowed her to “feel connected” in a way that made her rely less on her eyes. Through the process of familiarizing herself with a sonic response—but also co-designing that response with others while learning and moving with them over extended periods of time—the networked wearable DMIs allowed this dancer (and others) to attain a rather different sort of connectedness through bottom-up experiential approaches and embodied learning.

### Collective Enactive Learning

To make better sense of what this dancer is expressing, some remarks on the enactive (and earlier autopoietic) noncognitivist approaches to thought informing our design strategies should prove helpful. According to this approach, a living system enacts a “world,” a cognitive architecture, by means of its ongoing continuous activity (Varela et al., 2017). Cognition is tantamount to the process of life itself, “immanent in matter at all levels” (Capra and Luisi, 2014). In our workshops, the upshot is that the musical wearables adorning the bodies of the participants acquire such a function, just as refined sensory organs do in being shaped by a phylogenetic history of environmental coupling (Capra and Luisi, 2014). These architectures resemble “a patchwork of subnetworks assembled by a complex process of tinkering, rather than a system that results from some clean, unified design” (Varela et al., 2017). The ongoing processes of refinement we pursue in developing these instruments abductively, through participation and without *a priori* models, actualizes a key affordance of real-time media processing with interpreted software programming environments, namely that one must no longer contend with a delay between composition and sonic actualization (Thorn and Sha, 2019; Thorn, 2021a). Rather, continuous reshaping of a body of code through trial-and-error tinkering refines and symmetrizes the sensorimotor dynamics of embodied action and perception, which are undifferentiated in the enactive approach. DMIs can be built on the fly without models, leveraging participatory dynamics to create a situation of collective enactive learning. Hence, the previously mentioned dancer could allude to the development of a *collective sensorium*, as it were, as a processual outcome of participatory workshopping with real-time, abductively constructed wearable DMIs. Abductive reasoning, likewise, is the mode of critical learning taking place in transdisciplinary computational creativity (Filimowicz and Tzankova, 2017). This design space addresses an aperture in the scientific literature being incipiently explored by a variety of MOCO researchers and practitioners bridging the artistry of interactive dance systems with scientific interest in movement sonification for sensorimotor learning (see Bevilacqua et al., 2016; Giomi, 2020 for concise summaries of this research space).

## Design of Special Hardware and Software Kits for Collectively Playable Wearable Music

The multifaceted term “workshop” suggests a range of diverse activities, including discussion, exchange, interaction, as well as do-it-yourself (DIY) communities and citizen participation (Jo et al., 2013). Following precedent work in collective musical workshopping, we envisioned hosting workshops with movement-responsive musical wearables for expert dancers using aggregate sensing with rich and animating musical responses. This interest grew out of our previous collaborations combining sound and dance, and from our discussions about building more symmetrical relations among coding, choreography, dance, and musicianship. This work continues a line of research in responsive performance environments for dance with a long and formative history at our home institution, Arizona State University (Lovell and Mitchell, 1995).

### Wearable Sensor Kit

**Figure 2.**
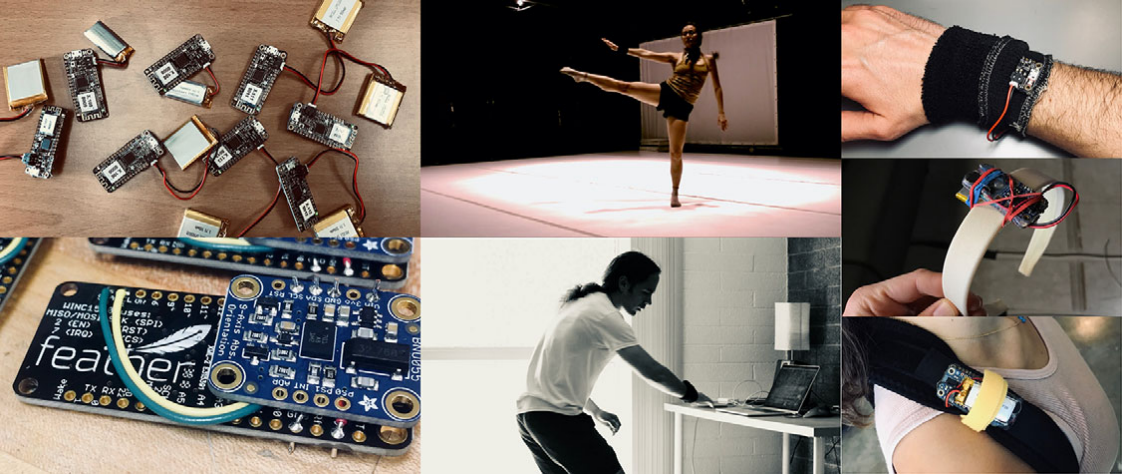
*Left:* the bespoke sensor kit. *Center bottom:* the first author using one of these sensors on his wrist, and (*center top*) the second author doing the same while dancing at the *Intelligent Stage* at Arizona State University. *Right*: some of the sensor placements we explored in the workshops.

The first author developed a wireless sensor kit based on a development board from Adafruit and an inertial measurement unit (IMU) from Bosch (BNO055) with 9 degrees of freedom (DOF) and on-board sensor fusion.^3^ The boards are powered with 400 or 500 mAh lithium polymer batteries and connect as clients to a high-performance wireless router tethered to a laptop using wired ethernet. The microcontrollers transmit Open Sound Control (OSC) formatted^4^ User Datagram Protocol (UDP) packets via Wi-Fi to the host server at a rate of 100 Hz, the full data rate capability of the Bosch IMU (Figure 2).

### Software Strategies for Collectively Playable Wearable Music

The host server runs an Ableton Live (AL) session and bespoke Max for Live (M4L) devices designed by the first author for synthesis, mapping, and aggregate statistical processing. AL is a popular commercial audio software capable of running as a standalone digital audio workstation or in tandem with an embedded version of the interpreted programming environment Max/MSP, an ambient data-flow software that has been the lingua franca of real-time media processing for several decades.^5^ The real-time audio processing effects available in AL can be combined with the lower-level and more adaptable analog-modeled visual signal processing of Max/MSP, which are programmed as individual abstractions or “patches” that wrap various methods of scaling, ramping, interpolating, and/or utilities for time-series analysis, event detection, random sequences, and so forth.

**Figure 3.**
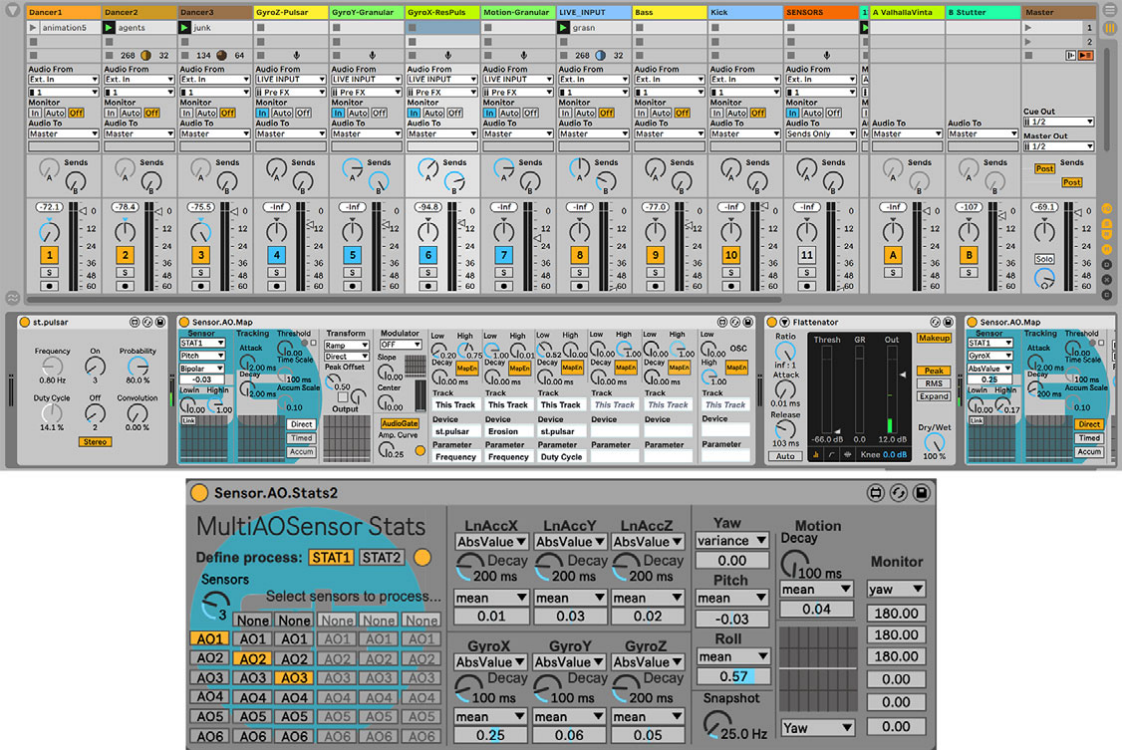
*Top:* an Ableton Live (AL) session with embedded Max for Live (M4L) patches. Vertical columns represent a bank of input channels as would be found on a traditional audio mixing console, with each channel providing amplitude gain, stereo panning, and routing options. The first three channels (*Dancer1*/*Dancer2*/*Dancer3*) contain pulsar synthesizers coupled to individual sensors worn by three dancers. Additional channels receive audio input from various synthesizers driven by collective mappings. In this figure, the channel *Gyro-X-ResPuls* is selected, revealing a corresponding audio signal chain in the lower third of the window. The signal chain moves from left to right, starting with a bespoke pulsar synthesizer followed by an instance of the specialized M4L mapping patch (*Sensor.AO.Map*) designed by the first author. In this case, an aggregate statistical feature, the mean pitch of the three inertial measurement units (IMUs), is selected and mapped to the frequency and duty cycle of the pulsar synthesizer, as well as an “erosion” effect (a short delay line modulated with filtered noise) that is placed further down the signal chain (clipped offscreen in this figure). *Bottom*: the M4L statistics patch designed by the first author, which allows aggregates of sensor streams to be defined and evaluated statistically and subsequently used as control signals by mapping patches.

We have found it to be difficult to design a collectively playable instrument from the ground up, but easy and efficient to adapt an individually playable instrument to collective playability—and quick if appropriate tools are available to perform the conversion. This is the primary thrust of the new utilities we present here. Prior to the workshops, the first author developed a set of bespoke M4L patches for quick construction of individually and/or collectively playable wearable DMIs.^6^ The primary M4L patch used for this, a parameter mapping patch that maps streams of sensor data to audio synthesis, requires initial selection of a wearable sensor (associated with a UDP port) and IMU DOF or related feature (Figure 3). The control signal can be shaped with a variety of arithmetic or logical operations. An individually playable instrument is then quickly converted to collective playability by replacing the selection of a single IMU DOF with a statistical measure of the same feature aggregated across two or more IMUs, e.g., by exchanging the pitch of sensor *n*, normalized to a range of −1 to 1, with the coefficient of variance of pitch among a group of sensors normalized to the same range. This mechanism mitigates the jaggedness of real-time digital composing, the “detachment” or “disruption in flow” that characterizes the intervallic and tedious nature of DMI development (Magnusson, 2009).

Using a visual interface, the mapping patch makes available a variety of basic signal processing operations, such as scaling and clipping to remove noise and define dynamic range, as well as timers, accumulators, attack and decay envelopes, and functions such as inversion or other wavetables to creatively reshape and texture the control signal. This signal can be convolved with pitch, roll, or yaw angle, sensitizing to a region of movement via adjustment of a center peak and sensitivity breadth.^7^ These simple operations present legible and potent transformations for workshop actors. Multiple mapping patches can quickly be instantiated in AL, allowing selection of additional features, signal transformations, and parameter mappings. In our design, the control signal from the mapping patch can be mapped to multiple parameters across the entire AL session, including global parameters in the master audio bus or signal chains associated with individual wearable sensors, which mitigates some of the linear design ontology of AL, a corollary of being modeled as a traditional audio mixing console.

In a typical use scenario, a mapping patch is used to couple 3D acceleration magnitude to the amplitude gain of an audio processing chain, which mimics the fundamental physics of an acoustic instrument by preserving aspects of energy continuity, while one or more additional patches are instantiated as modulation controls. This “supra-instrumental” approach, by which different gestural functions are abstracted from analysis of acoustic musical instruments—particularly gestures of “excitation,” “modulation,” or “selection” (Cadoz, 2009)—is a useful starting point for building new DMIs in this otherwise highly abstract design space. Figure 3 shows the fully developed AL/M4L session that developed and crystallized over the course of our four workshops.

### Strategies for Collective Mappings

**Figure 4.**
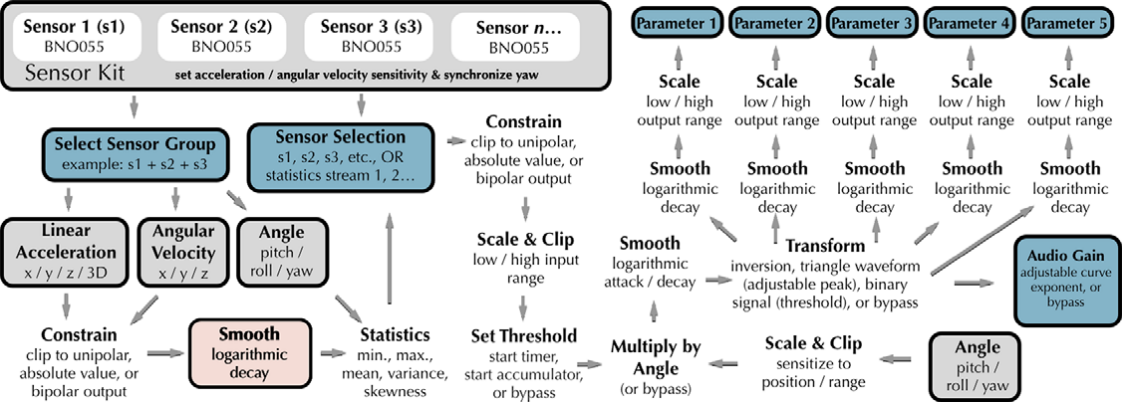
This diagram shows the signal flow between the mapping and statistics patches (shown in Figure 3).

Real-time windowed cross-covariance for correlated motion tracking of dancers is a well-researched technique (Aylward et al., 2006; Krzyzaniak et al., 2015). Decisions about window size create a tradeoff between the maximum time separation among the correlations and windowing latency. Similarly, the M4L patch we built for statistical analysis of data from multiple sensors defines a temporal region of simultaneity by applying an adjustable decay envelope to individual sensor streams prior to the aggregated statistical evaluation (Figure 3). From the perspective of our processual and practice-situated approach, the windowing tradeoff defining what counts as “simultaneous” is less a theoretical issue than a practical one we approach experimentally/experientially to investigate how modulation of the decay envelope affects the CDI dynamics. Simple statistical operations such as minimum, maximum, mean, skewness, variance, or circular variance for yaw angle, can be selected and mapped with this patch. Other operations are available prior to the decay envelope (Figures 3 and 4). Several statistical patches can be used simultaneously if multiple modes of processing the same sensor stream are needed, or to define and process different aggregate configurations of data streams.

## Wearable Music Workshopping for Expert Dancers

We now proceed to our description of the workshopping events themselves. During these events, we worked with seven dancers altogether, inclusive of the second author. Four events took place over the course of 2 weeks with 3–5 dancers attending each session. The authors attended all sessions. The other dancers were current or former students of the second author’s university courses, but not all of them had previously worked with each other. Workshop sessions lasted between 90 and 120 min, a constraint mostly imposed by the physical strenuousness for the dancers.

### Task-based Movement for Ensemble Thinking

**Figure 5.**
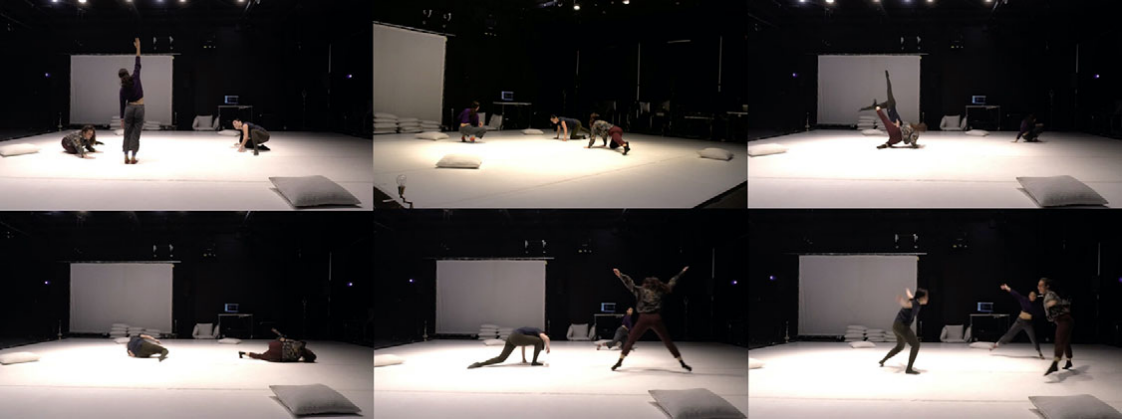
Still frames from a durational score (from *left to right*, *top to bottom*) showing (a) emergence of leading (b) followed by reintegration, (c) a novel motif followed by call and response, (d) introduction of a second motif, (e) and introduction of a third motif leading to (f) group unison movement. The video can be viewed at https://vimeo.com/374805505.

To guide the workshops, we found inspiration in the toolkit developed by choreographer Nina Martin for group improvisation, “ensemble thinking” (ET) (Martin, 2007). According to Martin, the goal of ET is to create “a performance language wherein creative choices made by the individual performer can be understood and acted on by the group” (Martin, 2007). Through group exercises, the system trains dancers into awareness of the divergence between their “microlevel” personal movement vocabularies (“composing in the kinosphere”) and “macrolevel” compositional concerns in group improvisation—simultaneous awareness of *what* one is doing with one’s body and *where* one is in time and space (Martin, 2007). This awareness is difficult to develop and maintain, especially with larger groups. ET uses exercises that promote simple decision making in the large group setting, facilitating quick, agile responses to the dynamic of emergent leading and following. We found additional motivation in the approach outlined by the Paris-based *ICI* research group, whose constituents investigate CDI experientially (Himberg et al., 2018). ICI’s design principles for the phenomenological study of togetherness include open-endedness of movement sessions to enrich the first-person perspective and emphasize process, minimization of visual/verbal support to highlight and activate kinaesthetic experience, and “elicitation in the shifts in the patterns of behaviors”—nonlinear, chaotic bifurcations experienced as qualitative shifts in affective dynamics (Himberg et al., 2018). A prosaic and straightforward example is the spontaneous concurrence that an open-ended improvisation is reaching an endpoint for the group (Figure 5).

**Figure 6.**
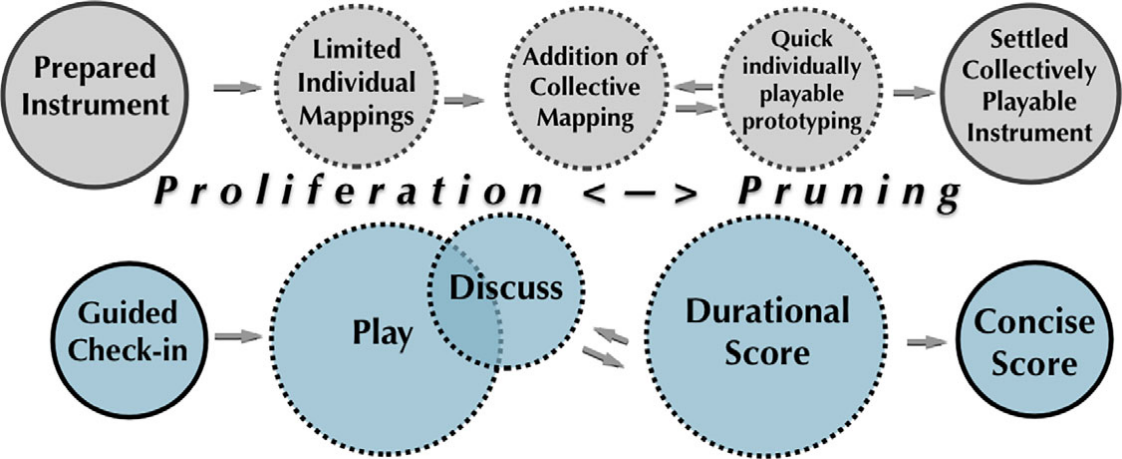
This diagram depicts the dynamic evolution of our workshopping *qua* structured improvisation: phases of proliferation and pruning, as well as parallel cycles of instrument adaptation and waves of learning by participants, mark iterative cycles of play, discussion, and exploration of durational scores. As in improvisation, there is a *felt energy* in the room vis-à-vis the learning and cohesion that have taken place, suggesting the potential of consolidation in a culminating concise score.

The workshopping structure was conceived and adapted by the second author, who initiated each session using a guided check-in to promote relational awareness, followed by periods of play/discussion using the networked wearable sensors, and exploration of various *ad hoc* scores she generated on the fly. We employ the term “score” here loosely to indicate a set of instructions that delimit the movement content of an improvisation and influence its evolution, typically by prescribing physical tasks to be completed (Buckwalter, 2010). “Straitjacketing” the movement vocabularies of dancers to limit their personal movement vocabularies not only attunes the dancers to one another, but also brings more awareness of the media response to their individual and collective movements and bodily orientations. The second author blended phases of open play, informal and more formal discussion, and use of durational or concise scores according to the didactic needs she perceived. (These terms are defined in Table 1, with examples.) Durational scores tend to evolve through several emergent phase changes. Macrolevel awareness is facilitated in durational scores by lack of predetermined cues indicating an ending, with spontaneous collective awareness emerging to conclude the event. Durational scores, moreover, are emphatically *loose*: the prescribed tasks and awareness exercises are *lightly held* ideas, proposed yet open to deviation by unanticipated intervening forces. Thus, despite the prescriptive structural dimensions of these scores, they remain open in a way that supports a focus on process. The waves of learning that build up through multiple iterations and cycles of open play, discussion, and durational scores, prepare dancers for a culminating “concise” score that is time-limited and more resolutely task-oriented, since it assumes that dancers have developed broad macrolevel relational awareness of the group, as well as an understanding of the sensing and media response features (Figure 6). This heightened awareness of time and task fulfillment during concise scores has a compositional purpose: it requires dancers to make quick and agile improvisatory decisions that realize these tasks before reintegrating into the group.

**Table 1.** Fluid workshopping methodology

Unit	Exercises
Check-in (5 min)	Guided awareness sessions and relational exercises: Breathing to promote full-body awareness. Guided imagery: air as viscous stuff interconnecting bodies. Perception of ecological relations to other creatures, earth, and universe.
Play/free discussion	Free exchange between playful exploration and discussion: Exploration of individual voicing/others’ voicings (i.e., mapping of movement to sound generation). Noticing activation of group inflection. Informal ad hoc oral discussion of aggregate sensing parameters.
Guided discussion	Guided formal discussion in which dancers: Share encouraging or stifling experiences. Inquire about or suggest new sounds, mappings, and ideas for scores.
Score: durational (15+ min)	Loose set of instructions + extended and open-ended time for nonverbal learning and development of relational awareness and full-body attention/intention: Emergent solos/duets or unison movement, flocking. Periods of serene movement punctuated by vigorous/exaggerated activation of musical wearables. “Straitjacketing” of movement vocabulary to pedestrian movement (e.g., standing, walking, turning, crawling, arm swinging; Martin, 2007; Buckwalter, 2010; refer to the video at https://vimeo.com/393178043 for an example). Periods of unison movement. Imagery of viscous or precarious stuff (e.g., moving through water, walking a tightrope or on a frozen lake). Adding or removing props from the stage.
Score: concise (3–5 min)	Strict set of tasks to be completed in short period of time: Assumes development of macrolevel awareness, including familiarity with media response. Creative resolution of scores through emergence of leading by individuals who solve prescribed tasks before reintegrating into the group.

### Progression of Sound Synthesis and Mapping Techniques

The first author sketched a collectively playable DMI that he brought to the first workshop as initial material. This instrument uses a variety of particle synthesizers that include pulsar, granular, and glisson instruments (Roads, 2004).^8^ Particle synthesis is especially useful in new DMI development for students without traditional musico-theoretical training, due to its thematization of physical features less freighted with music theory (e.g., density/spatiality vs. notes/harmony). Granular synthesis generates sound by indexing an audio buffer that is granularly dispersed by synchronous or asynchronous clocking of randomly selected playback positions, grain durations, sampling-rate alterations, or other algorithmically determined transformations. Alternatively, live audio input can be recorded into a circular buffer that is granulated and periodically overwritten with new audio input. In the basic instrument prepared for these workshops, extensive use of the circular technique was made: an 18-s audio loop composed by the first author, embedded with shifting musical harmonies, is continuously looped and played into a short circular buffer referenced by multiple granulators. Features of these granulators are in turn activated and modulated by the wearable sensors in real-time in synchrony with the playback of the loop, which gives compelling musical consistency, material variation, and forward propulsion. The loop can be replaced with a different sound file, preserving the formal structure of the instrument while immediately transforming its material and connotative qualities. Since the varieties of particle synthesis used in this instrument evoke ebullient, percolating, and continuously evolving sonic textures, we named it *mare vaporum*, “sea of vapors,” after the eponymous sea on Mars.

As we discovered, however, initiating a workshop with collective mappings is mystifying for the participants. It is much more skillful to begin with individual voicings that demonstrate clear relationships between individuals’ movements and sound. We quickly maneuvered to such voicings by coupling three pulsar synthesizers to the 3D acceleration magnitude of the three individual sensors, differentially spatializing them within the multichannel sound stage to improve learnability of the kinesics.^9^ Pulsar synthesis was selected due to its clear control structure for manipulating density and spectral brightness, as well as its continuous rhythmic and versatile timbral affordances. In acoustic instruments, spectral complexity and amplitude tend to be highly correlated, thus making for an adept design choice in inceptive DMI designs. Thus, coupling and scaling the 3D acceleration magnitude to amplitude, frequency, and duty cycle parameters of the pulsar synthesizer quickly generates a compelling and intuitively playable wearable DMI.

Once the dancers became familiar with the response and sensitivity of the individual sensors and pulsar synthesizers, we began experimenting with richer and more intricate instruments and collective translations. These instruments progressed through phases of adaptation and transformation, a process diagrammed in Figure 6. Omitted in this figure is the orthogonality of these processes, the gradual convergence of the different expertise domains as the participants become more attuned to the technical practices of the others. Moreover, the workshop itself has the character of a structured improvisation marked by periods of proliferation and pruning, a trial-and-error dynamic in which potentialities of the assemblage are teased out by assessing the outcomes of off-the-cuff experiments and seizing on the most palpable and expressive structures (Angelino, 2018). Proliferation proceeds rapidly as new ideas and mappings are quickly implemented and explored, blending experiments with both individual and collective mappings. “Pruning” indicates processes of selection as well as refinement and exfoliation. This dynamic plays out across multiple timescales and structural levels: not only at the level of the domains of individuals’ technical expertise (i.e., expressive movement, dance scores, sound mappings, and synthesis algorithms), but also vis-à-vis the 2 weeks of workshopping, which converges on a final concise score (described in Section 4.4).

To lay out this process as a sequence of steps goes against the grain of the dynamic they represent. Nevertheless, the following procedure captures aspects of the knowledge we acquired that can be brought to future workshopping events and research-creation:

1. Begin with some preliminary movement scores and mappings of movement to sound. Allow workshop participants to explore individual voicings.
2. Invite dancers to play with these preliminary voicings; discuss their experiences with them; vary, switch, and iterate voicings into more fleshed-out instruments mapping movement to sound.
3. Create a rich instrument that is individually playable, then exchange the individual mappings for the respective collective parameters.
4. Provide more open sets of movement guidelines for the participants to work and improvise with. The instrument behavior can be modified ad hoc. The collective can intensify the *felt experience* of the improvisation by gradually adding new sounds and sensitivity to different movements. Learnability of the kinesics can be balanced with dynamic modulation of the instrument.

### Wearable IMU Placement

One obstacle we encountered during the workshops is the surplus attention drawn to the wearable sensors and consequent congestion of group improvisations. Despite their diminutive size, wearable sensors somatically freight the bodies of the participants by drawing awareness to the location of the sensor, altering the first-person kinaesthetic experience of the body (Höök, 2018). We experimented with placing sensors on the foot, head, and shoulder. Distal placement strongly choreographs movement. Placement atop the head using a headband, for instance, encourages dancers to initiate movement there. Observing this, the second author spontaneously joined the group as a noninstrumented dancer who could relieve this congested movement by corporeally proposing less sensor-centric movement motifs. In technology-augmented dance, noninstrumented dancers can make choices or introduce motifs not driven by the presence or position of the sensor on the body or sonic response to movement. Dancers wearing sensors are relieved by this new space for choices that are less wearable-focused yet still wearable-influenced—they become receptive to “exterior” influences that may catalyze new intensifications (refer to the video at https://vimeo.com/373915236 for an assemblage of instrumented and noninstrumented dancers). Prior to the third workshop, we experimented with placement atop the right shoulder near the neck, which proved successful for the purpose of promoting unreserved full-body motion rather than intentional movement from the sensor in the form of distal initiation and limb-specific gesture. Proximal rather than distal placement of the wearable sensor is a more agnostic way of instrumenting dancers, and we propose that there are somatic reasons for this based on an understanding of fundamental core-distal connectivity, whereby by the navel “functions as a primitive center of control” (Bainbridge Cohen, 1993).^10^

### Culminating “Concise” Score and Collective Instrument mare vaporum

Over the course of four workshops, we progressively developed a coherent and nuanced collectively playable instrument (Table 2 summarizes its features). Since no dancer attended all the workshops (typically two of the four, apart from the continuous presence of the second author), this created an interesting dynamic, as dancers would return to an instrument that had developed further. The culminating event of each workshop is the realization of a concise score. In the final workshop, the dancers called this score “Pillow Score” due to their use of large pillows we found in the black-box theater space and incorporated as enriching compositional elements. During this final workshop, we also experimented with an unusual mapping that transformed the entire *mare vaporum* instrument, namely the coupling of group movement intensity (aggregate mean 3D acceleration magnitude) to diminishing amplitude and increased lowpass filtering of the entire instrument. This was easily implemented by adding a mapping patch and lowpass filter to the master audio bus in AL. As a result, a narrower range of movement activates the original parameter mappings, while excessive movement attenuates the response of the entire instrument, including individual mappings. Modification of the decay parameter of this inverted envelope, the rate at which it bounces back to an unattenuated state, strongly conditions the movement of the dancers by determining how gradually the instrument becomes sonically enlivened again (refer to the video at https://vimeo.com/393185524 for initial exploration of a shorter rebound, and to https://vimeo.com/375157582 for excerpts from “Pillow Score” showing a much slower rebound). This greatly intensifies macrolevel awareness: anyone who moves *too much* blunts the sensitivity of the instrument for everyone else, which instantiates a distinctive economy of collective intention and attention.

**Table 2.** *Mare vaporum* instrument

Feature	Statistic	Modulation	Description
3D accel. Magnitude	None: (indiv.)	–	Each dancer controls a pulsar synthesizer amplitude-convolved with looping samples of human voices. (Amplitude convolution is a frequency-domain operation that uses bin amplitudes of one sound to modulate the bin amplitudes of another.) Increasing magnitude is coupled to increased audio gain, pulsar frequency, spatial reverberation, and decreasing duty cycle from 50 to 1% to increase spectral brightness.
3D accel. Magnitude	Mean	–	Granular synthesis of looping audio buffer. Increasing magnitude increases granulation frequency. Grain length is fixed at 250 ms. Steep shelf filters roll off low and high frequencies.
Gyroscope Z	Absolute mean	Pitch (mean)	Increasing angular velocity is coupled to increasing pulsar frequency. Pulsar probability is fixed at 75%. Additional spectral effects. Aggregate mean pitch modulates duty cycle.
Gyroscope Y	Absolute mean	–	Granular synthesis of looping audio buffer. Increasing magnitude increases granulation frequency and boosts middle frequencies. Low frequencies are attenuated. The audio loop is pitched up and grain length is fixed at 50 ms. Automated panning.
Gyroscope X	Absolute mean	Pitch (mean) + 3D acceleration magnitude	Controls a pulsar synthesizer that enlivens a resonant filter bank. Unlike other mappings, aggregate mean pitch controls features that would more intuitively be coupled to motion: increasing pulsar frequency, decreasing duty cycle, and noise-modulation of a short delay line adding spectral complexity. An amplitude gain control at the end of the signal chain is coupled to aggregate mean 3D acceleration magnitude. (Figure 3 shows this mapping.)
Linear accel. *Z*-axis	Absolute mean	–	A distorted sinusoid waveform tuned to *E0* is triggered by group stomping when a predefined threshold is exceeded. The decay envelope duration is set to 300 ms. To make this sound more *gestural* rather than simply *triggered*, the magnitude of group stomping is coupled to additional down-sampling of the waveform, increasing its spectral complexity by degrading the signal resolution. Refer to the video at https://vimeo.com/393324437 for a stomping demonstration using this mapping.
3D accel. Magnitude	Mean	Yaw (circular variance)	Controls the amplitude gain of a synthesized bass tone. Yaw variance is coupled to spatial reverberation, spectral effects, depth of low-frequency oscillation (LFO), and rhythmic rate of a resonant lowpass filter. When yaw variance crosses a threshold, the bass tone cycles to a new note (*A0*, *E1*, or *E0*), triggering as the dancers move in and out of alignment.
Gyroscope Z	Absolute mean	Yaw (circular variance)	Controls the rhythmically synchronized triggering of a kick drum. Group yaw variance controls amplitude: high variance turns the sound off completely; less variance increases the amplitude gain. Sound generation thus depends both on group state *and* group movement. See the video at https://vimeo.com/393177774 for a demonstration of this mapping used in unison/flocking.

Below, we reproduce the second author’s instructions for the culminating concise score in the last workshop, “Pillow Score.” Tasks may occur in any order. As with durational scores, these are baseline instructions that dancers may ultimately choose to ignore or vary, their purpose being to generate a lexicon of motifs that orient dancers throughout the event by *conditioning*, but not *determining*, its concrete unfolding. This openness allows the actors to exercise their own artistry by following these motifs or generating novel ones on the fly. Likewise, emphasis on the concision of the score is more about conditioning the vigor and pace of activity and artistic decisions—the “felt time” of the event experienced by the body (Wittman, 2016)—and less about predefining a firm and objective time constraint. Instructions to embellish or revel in different tasks generate excitement and encourage introduction of novel movement motifs.

“Pillow Score” (three dancers)

1. A dancer is covered in pillows. The other dancers enter one by one.
2. The dancer emerges from the pillows.
3. Dancers revel in the activity of clearing pillows from the stage.
4. Dancers find and engage in moments of unison.
5. Dancers indulge in sensor play.
6. Two solo moments and one duet moment are allowed. Movement energy is vigorous in solos but more restrained in duets.^11^

## Collaborative Telematic Wearable Music (Remote Course)

### Practice-Situated Telematic Research and Collaboration

The first author consolidated materials and approaches from the workshops with dancers to prepare a distance learning wearable music course he taught in Spring 2021. The course explored networked, wearable, movement-responsive instruments to shore up and remediate the lack of shared social and tangible affordances in virtual classrooms. This work echoes precedent work in sonic interaction in mixed-reality environments using spatial affordances (e.g., Wozniewski et al., 2013) but is a unique approach. In the context of telematic learning, collaborative wearable music also presents an opportunity to address fatigue from tedious foveal/screen-based interaction, a problem taken up by other mixed-reality techniques (e.g., Montpellier et al., 2015). Can we interact without a screen using sound and rudimentary wearable sensors? Can these foster a sense of telematic togetherness by facilitating more spontaneous and embodied interactions? What is the minimum signal needed to feel connected, and how scalable are the techniques? We reviewed telematic sonic interaction design and reflected on problems with remote and augmented collaborative learning (Akçayır and Akçayır, 2017).^12^ Due to the pandemic, it was necessary to adopt off-the-shelf, inexpensive, agile tools that could be continuously adapted to different and evolving needs, rather than designing bespoke interfaces under more idealistic and controllable circumstances. One is hard-pressed to find circumstances more apropos than a global pandemic for practice-situated exploration of telematic, embodied togetherness using networked wearable media.

### Wearable Music Course Design

The wearable music course is conceived for epistemically and culturally diverse undergraduate students. Five students were music majors, with 14 others coming from design, film, informatics, animation, and electrical engineering. The gender balance in the course was more favorable than is typical of an “electronic music” course, with 7 students identifying as female and 12 identifying as male. Wearables were recognized early on in affective computing as trenchantly affective adornments due to their intimacy with the body (Picard, 2000). Designing and presenting the course around the experiential poetics of “wearable music” perhaps makes this research-creation more appealing to many students, not only by suggesting playful interaction rather than mastery, but also because of the body-centric metaphor, which potentiates a discourse around electronic and digital music contrasting with its normatively gendered and combative machinic tropes (e.g., of “triggers,” “controllers,” “commands,” “bangs”) (Rodgers, 2010). In the course, students acquire skills while working on individual projects, then explore collectively playable instruments later in the semester. The course is divided into four modules comprising two class sessions for 3 to 4 weeks each.^13^ This structure is outlined in Table 3.

**Table 3.** Wearable music curriculum

Unit	Student Project	Activities
Micro-controllers	*Wearable ideation and preliminary project proposal.*	IMUs, filters, calibration, Arduino IDE, M5Stick-C API, Wi-Fi networking, OSC/UDP. Concept applications: sonification for sensorimotor learning, connection without a screen, and embodied sensemaking.
Interactive Sound	*Wearable musical instrument employing one-to-many mapping.*	Wavetable synthesis, musical arpeggiation, granular synthesis, loop manipulation, sample “scratching,” variable delay lines, basic procedural audio (e.g., fire and jet engine), sound design concepts (spectral space, density, stepwise vs. continuous motion).
Space–time	*Design exercise: creatively implement windowing, event detection, entrainment, or a relational instrument using two IMUs.*	Connect multiple sensors, relational models (spread of arms) telematic relational models (split exciter/resonator), physical models of interaction (e.g., water pouring), collectively playable instruments (e.g., shared control of granular synthesis), event detection (sonification of footsteps on ice, snow, gravel), emergent coordination (telematic clapping, splashing in virtual shared pool), windowing to track activity over time (periodicity, coefficient of variation, moving average offset removal for linear acceleration), entrainment (tracking beat, density, stability), creative use of integration (“winding up” potential energy), phasing patterns.
Experiential Design	*Final project (open-ended).*	Weekly sessions alternate between labs for individual projects and collectively playable telematic wearable music experiments.

Abbreviations: IDE, integrated development environment; API, application programming environment.

### Low-Cost Off-the-Shelf Wearables

Students acquired two M5Stick-C wearables^14^—an off-the-shelf, inexpensive IoT platform—along with a wireless portable router, the TP-Link N300, which they used as an access point. Acceleration, angular velocity, orientation, and battery voltage readings are sent as OSC-formatted UDP strings and received in Max/MSP. The sensor is small and embeddable, with a built-in 80 mAh or 120 mAh lithium polymer battery, thin-film transistor (TFT) screen, and MPU6886 6-DOF IMU.^15^

### Wearable Music and Felt Time

**Figure 7.**
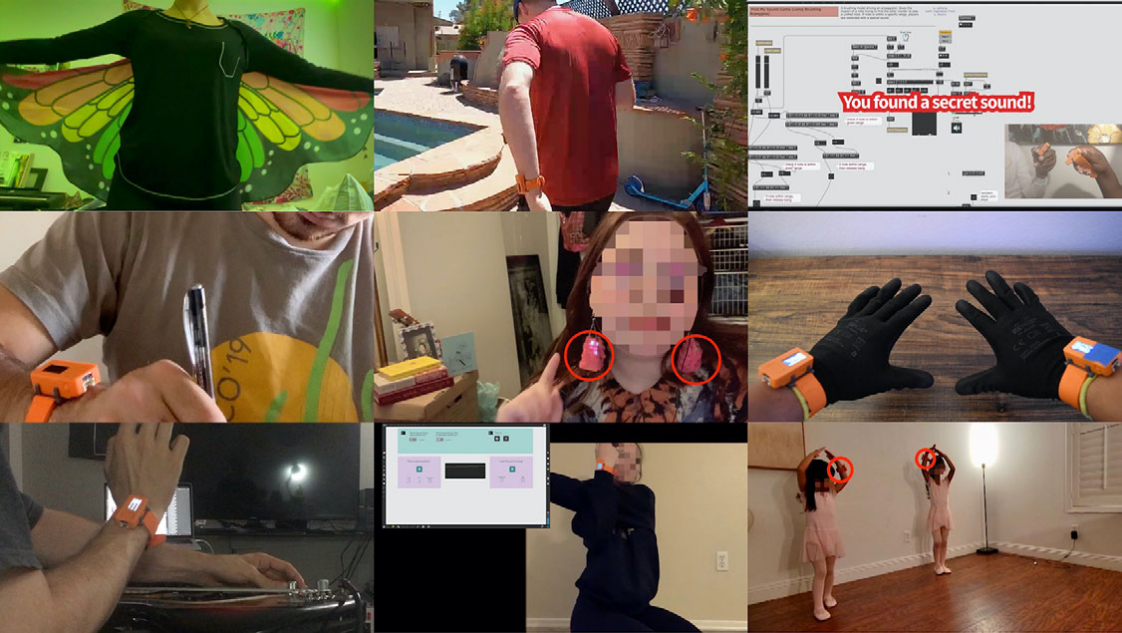
Students worked on individual wearable/movement/sound projects throughout the course. While preparing their final projects, we used class time to explore collectively playable instruments and surplus time for open labs. This figure shows a variety of creative final projects (from *left to right*, *top to bottom*): sonification of wings with two sensors, a project for encouraging fitness, a game in which users collectively search for hidden sounds, sonification of writing/drawing, movement-responsive “wind chimes” earrings, musical gloves, wearable augmentation of guitar playing, an automatic timing device for holding and changing yoga and stretching poses, and a student’s young relatives augmenting ballet poses and dance with wearable music.

As in the workshops with dancers, granular synthesis is fundamental to the sound design component of the course, along with physical modelling synthesis (simple excitation/resonance models), arpeggiating control of wavetable synthesis, and streaming input from students’ YouTube or Spotify playlists modulated with real-time audio effects from physical activity. Appreciation of atmospheric and rhythmical aspects of music (Böhme, 2017) are favored over western musico-theoretical analysis to create an inclusive classroom emphatic of “felt experience” (Gendlin, 1997). As with the *mare vaporum* instrument used in the workshops, cogent musical samples selected by students are parsed into short loops to create rich starting material encoding musical consistency and forward-impetus. Students reviewed empirical work evaluating the effect of musical agency on perception of physically strenuous performance (Fritz et al., 2013) as well as research on time perception as integration of bodily signals (Wittman, 2016). Taking this awareness into practice, students were encouraged to experiment with relations between time and movement, not only to modulate their perceptions of existing performance practices, but to invent new ones—musical wings, “wind chimes” earrings, or recovery of “lost” sounds through movement, are examples of enchanting projects students created (Figure 7). As a technical foundation for this work, students are shown how to accumulate 3D acceleration magnitude to drive the looping playback head position of a granulator, producing a granular time-stretching effect.^16^ For further inspiration, they watched documentation videos from the wearable music workshops, observing the experiments with the “inverted” mapping applied to the master audio bus of the *mare vaporum* instrument and the potent effect it had on the improvisation dynamics by modulating movement intensity and augmenting macrolevel awareness. This work lays a technical and experiential foundation for exploration of collectively playable wearable instruments later in the class.

### Jump-Starting Rich Explorations Using Parameter Interpolation

**Figure 8.**
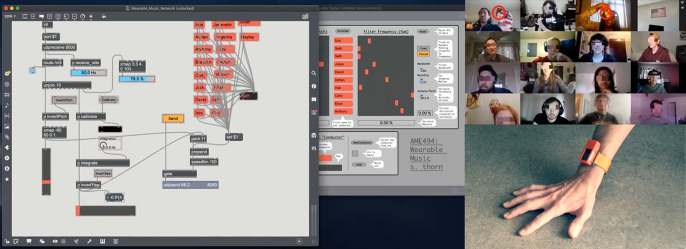
*Left:* screenshot of a Zoom session showing live editing of a Max/MSP patch in class to change the group’s instrument (see the video at https://vimeo.com/571056944). *Top Right:* zoom session showing first author and student “pouring water” back and forth between coupled wearable sensors. *Bottom right:* video still of a wearable digital musical instrument (DMI) demonstration shown to students using the pattr abstraction example patch (see Section 5.5; related video can be viewed at https://vimeo.com/571058528).

Wearable DMIs should render spectrally rich sound with meaningful physics. In this article, we have emphasized that DMIs become highly refined in evolving through exploratory abductive tinkering. To jump-start rich explorations in the wearable music class, students are provided a Max/MSP patch containing an encapsulation of a granular synthesizer exposing high-level features such as density, playback speed, and grain duration, along with spatial reverberation, lowpass filtering, and amplitude *tremolo* effects. Altogether, this offers a rich and parametrically dense suite of compelling tools for sound generation. An abstraction available in Max/MSP, *pattr*, can be used to quickly store and interpolate among unique parameter configurations. By mapping a sensor feature to the interpolation parameter, students can rapidly prototype wearable DMIs that implement a rich “one-to-many” mapping. As in the workshops, this design tool centers attention on experiential inquiry—the technical details can be fleshed out later, after student interest is piqued Figure 8.

### Relational-Experiential Media: Dyadic Instruments

**Figure 9.**
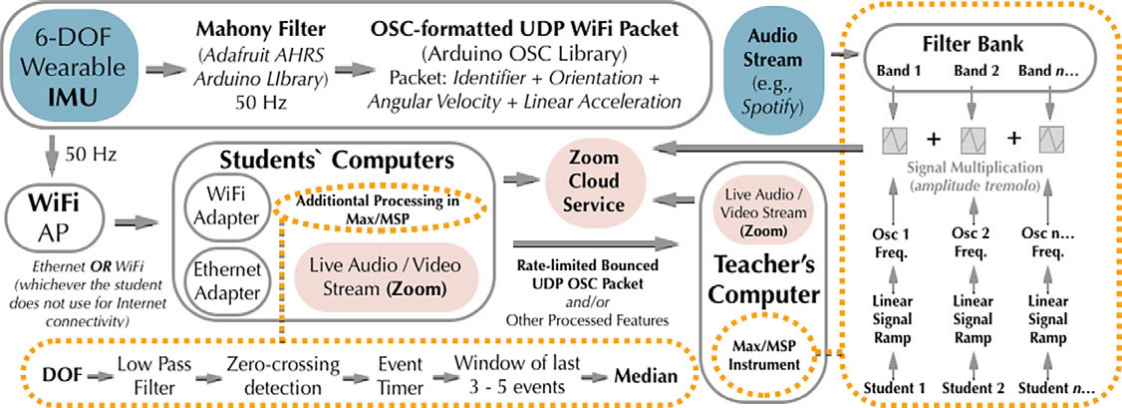
Network and audio processing topology of a simple entrainment instrument we explored.

We designate interactions involving two sensors as “dyadic,” reserving the term “collaborative” for the different interaction dynamics manifesting with DMIs involving three or more sensors. To introduce the unit on relational and telematic media, students were shown how to work with data from two sensors to calculate angle divergence, as well as integration of angular velocity to track the “winding” of one sensor in relation to another. The latter can control the resonant frequency of a virtual elastic band stretching between the sensors by implementing a simple Karplus-strong algorithm.^17^ Students then experiment with replacing one of the sensors with remote user input whose unanticipated activity may spur fresh design thinking by subverting expectations–often humorously–and intensifying affective engagement and discussion. While we explored peer-to-peer connectivity initially, blocked ports or other obstacles made this less feasible. Instead, collaborative telematic sessions are hosted centrally during class time by the instructor using a simple client-server model: students bounce OSC-formatted UDP packets from their wearables to the host server and receive broadcasted audio feedback via the Zoom video conferencing platform. The network topology is shown in Figure 9.

To provide a foundation for considering the relational dynamics of dyadic interactions, students reviewed a minimalist perceptual crossing experiment conducted by Auvray, Lenay, and Stewart, which explores recognition of intentional behavior by two isolated subjects whose movements are to be differentiated from static objects and moving lures (Auvray et al., 2009)^18^ This experiment eloquently reflects our interest in understanding minimum signals of connectedness. We imitated it by exploring remote entanglement of two networked wearable sensors whose pitch angles are compared using an inequality operation. Logical transitions generate an audible “click.” We embellished this by using different sounds for rising/falling logical transitions, such as different musical tones or resonant lowpass filtered noise sweeps that glide up or down in the direction of the transition. To enrich this model, we explored continuous “brushing” that replaces the discrete inequality calculation: absolute difference of the pitch angle of two sensors is inverted and scaled to create a region of sensitivity reaching a maximum value when the pitch angles are equivalent. This value scales the amplitude of clicks generated when activity occurs within the sensitive region. Subsequently, students learn rudimentary physical modelling synthesis by using these unit impulses to excite a bank of tuned resonant filters.

Students found these models conceptually intriguing in terms of the perceptual, networking, and synthesis concepts they introduce, but disorienting as an interaction paradigm. The multiple degrees of freedom of the IMU create mystifying kinesics for the students, leading to feelings of unpredictable responsiveness. As an alternative, we conceived of a relational physical model in which two remote sensors continuously “touch,” as it were, reminiscent of contact improvisation dynamics. Students were able to implement this idea using the physical modelling principles they had previously learned: one remote sensor performs an excitatory function (transient clicks) while a second performs a modulatory function that changes the resonant properties of the resonant filter bank. Students found this to be a more favorable interaction paradigm, so we extended it into other experiments, such as a 64-band frequency-domain equalizer that can be shaped with yaw and pitch angles affecting band selection and gain, respectively. The more continuous affordances of these interactive designs yield a better “grip” on the interaction, which we found to be prerequisite to any meaningful experience of telematic togetherness.

### Relational-Experiential Media: Collaborative Instruments

The “Global String” installation by Atau Tanaka demonstrates an intuitive conception of a resonant corpus shared between remote locations with continuous responsiveness, such that any number of individuals may collectively strike and shake the instrument (Tanaka and Bongers, 2002). We explored a similar idea by dividing the class into two groups on opposing sides of a “tug of war” game. Angular velocity of the *z*-axis of the gyroscope from all networked sensors is integrated using an accumulator running on the host server mapped to one or more audio synthesis parameters. As one group tries to drive the accumulated value higher, the other attempts to pull it lower. As an interaction paradigm, this is a helpful model to start with, insofar as individuals retain a sense of efficacy in the interaction as the accumulated value moves above or below zero—even as the number of participants is scaled up—whereas this is often not the case in larger group interactions. On the other hand, this interaction affords little relational awareness of *how* one is involved with the others. In other words, we experienced the same tradeoff between knowing *what* one is doing and *where* one is that afflicts CDI. It was helpful for students to consider the crossover with CDI approaches. This allowed us to begin articulating critical affordances for success in collaborative telematic wearable music.

As we learned from choreographer Nina Martin, the complexity of large groups tends to overwhelm the efforts of individual actors, multiplying the need for simplicity in improvisations (Martin, 2007). To explore this problem, we used simple models that we progressively scaled up by increasing the number of participants. For instance, we explored entrainment as a potential solution to the latency richness of telematic music. Students run Max/MSP patches that calculate the median of a sliding window of time intervals between IMU zero-crossings. As a participant moves the sensor back-and-forth in a periodic manner, the windowed median is sent to a host server that generates responsive sound by applying amplitude *tremolo^19^* to an individual filter within a tuned filter bank. Multiple students may be involved, with each remote sensor coupled to an individual *tremolo* rate of a different filter (Figure 9 shows this instrument topology). We discovered that, as individuals entrain the system, the system entrains *them* due to the strong sonic feedback signal, circumventing the latency problem. As the number of participants is scaled up, however, the bandwidth of the filters must decrease as new filters are added, and it becomes harder to perceive any one individual’s effect on the instrument—such is the zero-sum economy of spectral bandwidth. Another problem with this instrument’s design is the pre-schematization: individual sensors are rigidly coupled to different filters, a rather different paradigm than Tanaka’s string or Cage and Cunningham’s epochal responsive theatrical stage. At this point, the students in the class have accumulated enough experience that we can begin to articulate some guiding principles for collaborative telematic wearable music. Our first principle is:

1. *Unschematized interaction*: instruments should be adaptable to changing numbers of participants and continuously responsive to gesture and unanticipated forms of play.

We tried implementing this principle by redesigning an entrainment-based DMI that could accommodate varying numbers of participants without requiring rescaling of the instrument (e.g., filter bandwidths or other parameters). On the host server, a sound file is played back at 16 different sampling rates according to a three-note ascending scale (fourth, fifth, and octave), creating 16 individual but collectively harmonious “voices.” Amplitude *tremolo* is applied to each voice individually at intervals set according to a subharmonic ratio starting from 1 Hz, continuing by increments of 0.25–4.75 Hz. Students transmit the same periodic zero-crossing information as before, with the host server now rounding these values to the subharmonic intervals. These packets are routed into leaky counters coupled to individual voices, increasing their amplitude gain as packets accumulate. The idea is that sustained periodic movement of the sensor will excite the corresponding “resonant” frequency of the instrument, while amplitude *tremolo* provides entrainment feedback. Importantly, any number of students may participate at any moment, fulfilling our first design principle. While presenting an intriguing conceptual model alloying entrainment and resonance, it was difficult for students to perceive a relation between the entrainment and the sound generation. The situation became intractable when more students were involved. One of the difficulties is the narrow bandwidth of the individual “resonators,” which are narrowly spaced by 0.25 Hz intervals. This makes it challenging to deliberately sustain activation of a particular voice due to the complicated structure and slow response to students’ movements. Although we abandoned this model in the class to move on to other experiments, a simpler instantiation with fewer voices and wider bandwidths may be a good starting point for developing a better instrument. In any case, this led us to formulate a second principle, already well known in telematic music:

1. *Accommodation of latency-rich networks*: interactions should be based less on precise timing, perhaps by placing more emphasis on sensor orientation as input. However, entrainment paradigms may still work for simple interactions.

This second principle drew us to examine telematic orchestra projects. Musical works performed by these groups are commissioned to accommodate latency rich environments by requiring less precise timing (Rofe et al., 2017) and by construing network latency as a musical affordance—a propagation medium—rather than unequivocal privation (Chafe, 2009). As in traditional orchestras, a conductor helps the group to synchronize visually—even in concert halls, this visual aspect serves an important function in dealing with the latency of the auditory channel (Blesser and Salter, 2009). In our case, we adapted these features by designing a collaborative instrument requiring less precise timing, the central feature of which is use of an *auditory* conductor, which was suggested by a student.^20^ The instrument generates an ambient soundscape based on granular synthesis of a short loop passing through a bank of 10 resonant bandpass filters with equal energy distribution across the audible spectrum. *Q* and gain parameters of the 10 filters^21^ are coupled to the pitch angle of individual remote sensors, while yaw angles of these sensors are coupled to linear panning and different preselected frequency ranges for each filter, manipulated using stepwise motion to enhance the feeling of “grip” via this latching. Among the 10 participants, one is selected to be the auditory conductor, with the sensor yaw and pitch angles additionally controlling the amplitude and panning of a spectrally distinctive voice that is created by pitch-shifting the generative sound down by one octave. *Tremolo* and lowpass filtering of this voice are also controlled by the orientation of the sensor to make it more dynamic and expressive. To hyperbolize the spatiality of this voice, binaural panning is used to help further differentiate it from the other voices by additional spatializing cues as students listen through their headphones. As the conductor voice moves, students are asked to track it with corresponding movements. As everyone moves across the stereo field, the spectral quality of the instrument changes as the filter frequencies are all pulled up or down in tandem. Finally, the variance of the yaw angles is calculated: as variance decreases, the gain of an additional voice is increased, which is generated by a synchronous granular signal capture buffer with playback pitched up several octaves by increasing the sampling-rate. (Refer to the video at https://vimeo.com/571056944 for documentation of the in-class development process, including student suggestions and feedback, on the fly adaptation, and collaborative playing. Note that not all students elected to have their camera feeds on).

Among the instruments we had previously tried—dyadic and collaborative paradigms alike—this one received the most favorable response from students, who expressed a general feeling of ensemble (“it feels really collaborative”—see Appendix A) and engaged with the instrument for a significant period. Our exploration of these collaborative instruments mirrors the use of durational scores in our dance workshops: there is a mixture of verbal and nonverbal exchange, implementation of small control changes, and exchange of individuals’ roles in the group. CDI manages collaborative complexity by using task-based scores that skillfully condition distributions of leading and following. Orchestras, by contrast, typically employ a fixed leading position and top-down organization. Our instrument explores spontaneous exchange of the leading auditory conductor position, bringing humor, differentiation, surprise, and anticipation to the collaboration. For instance, at one point the first author noticed that a student was either struggling to align with the others or had simply stopped paying attention. In response, this student was given the conductor role, a solution which also sharply reattuned him to the spatialized ensemble that was now following his movements. Despite the fixed coupling of sensors to individual voices, the relative success of this instrument suggests additional design principles derived from the discussion with students:

1. *Leverage leading/following dynamics*: to manage the complexity of increasing numbers of participants, employ this paradigm to generate feelings of collaboration. This makes the interaction more compelling by potentiating sudden playful transformations of the group dynamic, which also creates a sustaining element by making the interaction more engaging for longer periods of time.
2. *Encourage macrolevel awareness*: to facilitate this, clever parameter mappings can be devised that respond to measures of alignment, group synchrony, or other events. (Note the emphatic reaction to aggregate orientation variance sonification both here and in the dance workshops).
3. *Rich sound*: more intricate, composed, evolving soundscapes steered with higher-level parameter mappings make for more engaging instruments in collaborative telematic wearable music, where parameter mappings must be kept relatively simple to remain palpable and effective. Simple mappings do not require simple sounds, which will quickly lose novelty.

Further consideration might be given here vis-à-vis tradeoff of the auditory conducting position by algorithm: not merely by chance, but based on the contingent activity of the ensemble, perhaps by employing a game-like structure that further engages students’ interest and intention/attention. At the same time, a performative and expressive understanding of play discloses how the meaning of a game derives not from design intentions but from what players actually do (Sicart, 2011)—a good reason to adhere to our first design principle, which calls for less schematized instruments that refrain from embedding strong goals or interactive intentions. Task-based scores may be explored to furnish the leading/following dynamics and shore up macrolevel awareness, so that the instrument can remain less schematized and more continuously responsive and resonant. Continuing this line of thought—looking for ways beyond the algorithm to intensify a feeling of collective togetherness—we can add two additional design principles, the first of which we discovered fortuitously when a few students in the class were already streaming packets from their wearables prior to any speech or video streaming.

1. *Prioritize the instrument*: before initiating other forms of telematic engagement (video and speech), explore the instrument as the sole means of interaction. This has a remarkable attuning effect on the interaction, renewing the uncanniness of the telematic.^22^
2. *Encourage embodied interaction*: if students are willing, have them place their sensors proximally rather than distally to encourage full-body movement. This enhances senses of spatiality/sociality and dampens fixation on manipulation of the sensor itself.

## Conclusion

The instruments we explored in the wearable music class and workshops were engaging, in part, by virtue of the processes through which they were collectively enacted. Student reviews compiled at the end of the course suggest that this participatory dimension challenged their perception of the educational context in a favorable way. As one student wrote in the anonymous course reviews, “It felt like we were not in class, but were a think tank of sorts, which was very refreshing.” It is significant that fewer than a third of the students in the course were music majors, with most coming from other disciplines or academic departments, and that there were no prerequisites for technical competencies. The objection could be raised here that, considering the minimal criteria for enrollment, given the somewhat technically rigorous and specialist content of the course, how could the outcome be more than a “tokenistic creative agency”? (Tanaka and Parkinson, 2018). An important factor is that the students had the benefit of 12 weeks of prerequisite projects and individual learning before reaching the final month of the course in which we explored collectively playable wearable DMIs together. Co-design does not always have the benefit of such drawn-out exchanges and learning. As for the dance workshops, a less sympathetic reader could emphasize that participation is thin vis-á-vis the sharing of technical competencies, but this overlooks the positive outcomes reported by the participants, while misplacing the emphasis on acquisition of preexisting technical competencies, whereas the salient generative dynamic in participatory design resides in the encounter of different tacit and embodied knowledges productive of *new forms.* Indeed, participatory design is sometimes viewed as a technique for drawing out tacit knowledge (Spinuzzi, 2005). As design theorist Anne Balsamo writes cogently, “diversity among design participants is generative…because people embody different sets of assumptions” (Balsamo, 2011). Here again, we invoke the notion of the rich and diverse, heterogenous “assemblage” paramount to both the MOCO ethos and transdisciplinary research-creation more generally. In the crisp synopsis of MOCO researcher Jan Schacher:

Research about movement-and-computing occurs at the intersection of several disciplines and perspectives. They meet and are mixed in ways that are less the result of deliberate choices and conscious engagement and are more contingent on the background, schooling, and practice of each actor. (Schacher, 2018)

Tacit embodied knowledge is the grist in the mill of practice-situated research. In the dance workshops and wearable music course, we built up collectively playable wearable instruments through co-design processes with epistemically diverse participants and movers in vibrant and complex assemblages. We deliberated verbally and corporeally, collaboratively tinkering with nuances in the signal processing, while observing how certain changes affect and vary how we play together and inform our sense of togetherness. In the process, we learned and adapted, orienting ourselves to novel feelings, sense, and sensations. With somatic and sonic finesse, we engaged with each other by proposing motifs with our bodies and with the new sounds those bodies produced, coupling movement to the sonic in ways that evoke friction, resistance, precarity, fickleness, pressure, or vibration. Although practice-situated research disfavors hard idealizations, explicitness, and discreteness, inasmuch as these would blunt the experiential phenomena, these inquiries are precisely *experimental* to the degree that the results they produce condition the dynamics of the collective action in *reproducible* ways (Sha, 2016). In both the workshops with the dancers and wearable music course, we were able to elaborate designs for networked wearable DMIs that affect our sense of togetherness, yet we are only scratching the surface of those potentialities, and we hope that our experiences will prompt others to pick up from where we have left off.

In summary, our design process benefited from the enriching complexity of movement styles and histories brought by the various actors (dance, yoga, violin, and sports), their divergent epistemic cultures (engineering, performing arts, and computer science), as well as the material aspects of the physical or mixed-reality spaces (pillows, latency, and headphones), and the constraints of the wearable technologies themselves (their tendency to draw a surplus of attention, for instance)—material flows that surface and are followed by intuition and chance. This assemblage, the ecology of practices exemplary of MOCO research-creation, animates a design situation that is new. By drawing on different embodiments and divergent practices, there is fresh potential to steer away from well-worn and unobserved disciplinary grooves. Moreover, this way of doing things will not forego the possibility that a solution may emerge in advance of a problem, a salient aspect of the abductive process. A fine example is the experiment with the collective “inverted” instrument, which surprised us in its manner of delicately attuning movers’ intention/attention to the others. There are certainly no acoustic instruments that get quieter the more intensively they are played—this is precisely the enchantment afforded by the DMI design space and process. The skillful intuition (and chance) involved in that development not only comes from the generative collision of practices, but from the incremental refinements enabled by lower-level signal processing and abductive trial-and-error approaches responsive to movement *tout court.* In this way, we arrive at refined DMI designs, using networked wearables, by following practice-situated “intuition in action” (Deleuze and Guattari 1987).

## Data Availability

The full collection of supplementary videos can be viewed here: https://zenodo.org/record/5762453.

## Footnotes

1 Early in the twentieth century, Martin Heidegger elucidated the phenomenological structure of intentionality (*intentio*) by leaning on its non-dualist etymological sense, “directing-itself-towards” (Heidegger, 1985). According to artificial intelligence critic and Heideggerian commentator Hubert Dreyfus, “Heidegger accepts intentional directedness…but he denies that intentionality is mental” (Dreyfus, 1991). Another important thinker in this context is Maurice Merleau-Ponty, a philosopher of embodiment whose description of “motor intentionality” depicts the body as being “already mobilized” in pre-reflectively undertaking familiar tasks (Merleau-Ponty, 1962).

2 To use dance-philosopher Maxine Sheets-Johnstone’s suggestive formulation, in pre-reflective movement we are dealing with an experience of “thinking in movement” (Sheets-Johnstone, 2011). From a human evolutionary perspective, the corollary view, as expressed by archaeologist and anthropologist André Leroi-Gourhan, is the possibility that “mobility [is] the significant feature of evolution toward the human state” (Leroi-Gourhan, 1993). “There would seem to be sufficient documentation to demonstrate that the brain was not the cause of developments in locomotory adaptation but their beneficiary” (Leroi-Gourhan, 1993).

3
http://adafruit.com

4
http://opensoundcontrol.org

5
http://ableton.com; https://cycling74.com

6 These patches were adapted from the first author’s augmented violin practice (Thorn, 2021a). See the video at https://vimeo.com/336510223 for earlier documentation of him experimenting with adapting violin bowing sensing to full-body movement practice. For use of this instrument by the second author in dance practice, refer to the video at https://vimeo.com/336897086. These formative experiments motivated our interest in hosting “wearable music” workshops.

7 In the NIME literature, this style of parameter mapping is referred to as “many-to-many” or “many-to-one” mapping (Hunt and Wanderley, 2002). “One-to-many” is another approach. These multifaceted mapping topologies stand in stark contrast to rudimentary one-to-one mappings used in early DMI design by more closely resembling the complex behavior of acoustic instruments.

8 In computer music theory, particle-based synthesis is contrasted with wave-based synthesis techniques such as wavetable or frequency-modulation synthesis. The wave/particle distinction is sometimes suggestively evoked in this literature in analogy to quantum physics. Pulsar, granular, and glisson synthesis are varieties of the basic technique. Granular and glisson synthesis generate time-varying sonic textures by overlapping short sound events that individually last between hundreds of microseconds and tens to hundreds of milliseconds. Glisson synthesis is more idiosyncratic; this technique generates brief tones that that sweep higher or lower in pitch, sometimes converging on or diverging from a fundamental frequency, or in other formations, yielding organic-sounding textures. Pulsar synthesis generates pulses or tones that emulate classical electronic synthesis, but with more powerful digital control techniques. The first author has explored an implementation of polyphonic pulsar synthesis to generate rich textures in other work (Thorn, 2021a).

9 The basic unit of pulsar synthesis is a “pulsar,” a sound particle consisting of an arbitrary waveform followed by an interval of silence (Roads, 2004). In the implementation we worked with in the workshops, we used a simple sine function as our wavetable and a Blackman curve as the amplitude envelope.

10 From this somatic perspective, it is also interesting to note how the second author, whose movement style is shaped by a long professional career of dance and somatic work, implicitly brought this perspective to the workshops, whereas the first author, informed by 25 years of violin playing, fostered a distal perspective to expressive movement.

11 Duets emerge in a variety of ways in dance improvisation. Initiation may occur with tacit visual contact or noticed similarity of movement quality. In our sensor-augmented workshops, sound often instigates this contact. Duets may also arise when a second dancers “steals” a solo by joining in, or when a dancer approaches another and gestures physically and/or with sonification to suggest an intention to join. Instructions about movement energy attune dancers to the effect of movement on the collectively playable DMI, encouraging them to explore the thresholds of the system, which may catalyze other motifs or task initiations.

12 Much research has been conducted on designing systems for synchronous remote collaboration in the long-standing computer-supported collaborative work community (CSCW), and more recently with augmented reality, virtual reality, and mixed reality (AR/VR/MR) techniques. CSCW explores design of persistent contexts for activity by considering differences between peripheral and focal awareness, the possibility for impromptu encounters, usability through spatial metaphors, and telematic reciprocity—a symmetrical sense of presence among the collaborating individuals or groups (Benford et al., 1998). Along with social and technological affordances, computer-supported collaborative learning (CSCL) also considers educational affordances, in particular those characteristics that enable specific types of learning to occur (Kirschner et al., 2004). Other collaborative technologies have been designed for convivial personal or familial engagement, including telematic dinner parties (Barden et al., 2012) or devices such as the “FamilySong,” which is designed for remote synchronous music sharing with intergenerational family members (Tibau et al., 2019). These examples use specialized bespoke interfaces such as networked turntables, tabletop video projection, touchscreens, and radio-frequency identification (RFID) technologies, which may have considerable expense and require much time for design and deployment.

13 The course site is publicly available: http://wearablemusic.net

14
https://m5stack.com/

15 Nominal IMU values have significant offsets causing drift. Averaging those offsets on power up and subtracting them from subsequent readings while timing the main loop to 50 Hz works well with the Mahony filter available in the Adafruit AHRS library we used, improving yaw drift, steadying UDP streaming, and extending battery life.

16 “Time-stretching” refers to the preservation of pitch content during manipulation of playback speed.

17 The Karplus–Strong algorithm efficiently implements a waveguide using a short feedback delay line equal to the length of the period of the waveform. A burst of noise into the delay line becomes periodic, simulating a plucked string sound.

18 In the experiment, each subject mouses along a strip tracking the contact point. The interface generates a feedback pulse whenever the participants’ contact points cross one another. A fixed object is also present that generates a pulse when crossed, as well as a moving “lure” coupled at a fixed distance to the mousing point. When participants get a pulse, they backtrack to scan the area again. If this point is indeed the contact point of the other participant, coupled oscillation behavior tends to result, and the participants can recognize their co-presence.

19
*Tremolo* refers to low-frequency periodic variation of amplitude.

20 See Appendix A for an excerpt of the Zoom chat file.

21
*Q* describes the focus of a bandpass frequency response around a center peak. A high *Q* creates a resonator that continues to oscillate/ring after it has been excited. As *Q* increases, spectral bandwidth decreases, yielding energy loss. Thus, coupling together *Q* and filter gain is an intuitive parameterization.

22 Consider the experience of playing Ge Wang’s celebrated iPhone app, *Ocarina*, with unknown others far away: “There are moments in Ocarina’s globe interaction where one might easily forget the technology, and feel a small, yet nonetheless visceral, connection with strangers on the other side of the world” (Wang, 2014).

## Appendix A

Verbatim excerpt of the Zoom chat log during a networked ensemble experiment.

**Table:**

00:30:52	Jack: Pretty fun
00:31:02	Hayley: the sounds are very cool!
00:31:07	Isabel: Cool way to interact with others
00:31:21	Ada: I liked it a lot. I really did feel like I could hear myself
00:32:34	Adrian: okay i got mine to work now and i see myself
00:32:42	Vincent: lol
00:34:14	Jack: I was just doing figure 8s
00:34:36	Patrick: I thought it was a cool concept when you were trying to conduct the ensemble, and they had to respond to directions, or a group goal. It made me wonder if there could be a way of conducting the ensemble using the watch, without using the screen for interaction or feedback.
00:35:46	David: I was accidentally the fake Seth for a while there
00:37:13	Adrian: You could do one where the users (us) do not know what were doing then later on let us know what kind of moments effect what and see how the sound differs
00:38:33	Adrian: this is fun
00:38:38	Ada: it feels really collaborative
00:39:34	Julian: Yeah!
